# Inflammation

**Published:** 1861-06

**Authors:** W. H. Atkinson


### INFLAMMATION.

BY W. H. ATKINSON.

The much discussed phenomena of this intricate subject, usually has but tended to tie us fast to some author's perception or conception of this interesting effort of the life forces to maintain a due equilibrium in the system, or reject tissues so broken as to be unfit for repair; or so to befog us that we are worse off after reading the early authors, than we would be if we came to the investigation with a free mind, because without false record—not, however, to him who recorded it, for no doubt if the exact conception that occupied him was embodied in the record, instead of being false to the perception of the inquirer, it would be demonstrable fact. Here, as everywhere, we are at loggerheads in definition, not in manifestation of the mutations of the vital actions. Were I to pursue the line of march so well gone over by the astute and learned, the definition of what is meant by the term would first occupy my thoughts and stimulate my endeavor. But the merest tyro will perceive that if we had a clear comprehension and satisfactory definition of the term, I should be justified in at once laying down my pen, after simply writing out this concise statement, whatever it might be, for the use of all who find themselves in need thereof. The old "To burn" definition would as properly belong to the changes wrought upon food to fit it to become a part of the system, as to this same action, when occurring out of the "primæ viæ" for the safety of the organism; as in all forms of erythema phlegmons, abscesses, ulcers, etc. For all these are but so many modifications of a true burning (changing by heat) of the various constituents of the body, their inflammable quality being necessary to fit them for its use.

All these being but forms of digestion in the little and local sense, sustaining the molecular life that in the aggregate and proper correlation constitute systemic life.

What practical advantage can accrue to us from a definition that begins only *after* the foundation is surely laid, and its important initiative already taken?

In severe cases of inflammation, a close observer will be enabled to perceive a distinct stage of preternatural coldness, "chill," before any increased heat ("pyrexia?") *can* manifest itself. If, therefore, this stage is apparent in *all* clearly *marked* cases that have ever been noticed, is it not pretty safe ground to assert that this is that very initiatory step, to elipse which, will also annihilate all its sequents? To my perception this is a demonstration!

What remains to be done is to ascertain exactly what constitutes this so called "chill," which to me is proven by the rationale of all the cases that have ever been observed, of which we had the treatment intelligibly delineated.

*Syncope* is one of the most prompt remedies for this state, in its early or more advanced stages. Why? Because the "vis-a-tergo" is taken off for a time, and the congestions allowed a space of uninterrupted *nerve* influence, without the modifying and retarding action of a disturbed circulation. What is denominated "chill" is but the sensible heat being withdrawn for a time.

I will here premise that we can not create or destroy caloric, the real agent whose modifications appear in *chill* and *heat*, expressions signifying the *latent* and *free*, or *sensible* state of this agent, caloric. The mode of bringing caloric from one state to another, whether from latent to free, or from free to latent, is, beyond all doubt, a molecular action of the bodies capable of holding it in a latent state in one molecular condition, and possessing a less degree of capacity thus to hold it in another or changed molecular constitution. Free always seeking the latent state spontaneously when this molecular action ceases to be repeated, by the law of contact with conductors, substances varying in capacity to convey the thermal current, good conductors quickly, and poor ones slowly establishing the equilibrium, by distribution, or by the free assuming the latent state again.

No tissue can be properly said to be in a state of inflammation until after a loss of the equipoise of the nutrient forces has laid the foundation for this peculiar manifestation thus denominated. This brings us to a point where we may well question whether this action be a cause or an effect. If it be but an effect, it can not be said to be a disease "per se," which will make it imperative for us to look behind this for the true cause, disease, that only takes the inflammatory method of expressing itself.

If, then, this loss precedes all the more obvious signs of disease, is it not quite fair to assume that the disturbing force is finer and more occult than the usual phenomena of inflammation? It is no difference how near we may come to this process by any sort of means, if we do not have the necessary constituents present—it can not be said strictly to exist. I know very well that plethora and bad nutrition, from whatever cause, is said to induce inflammation; but such methods of investigation only paddle about in shallow and narrow channels or pools, the least motion in which raises such a cloud of sediment as to prevent continuing the research for a time, till this debris has had time to subside. Let us at once go to the bottom of the matter, and assert that any departure from the purely healthy standard of nutrition is in fact but a degree of disease that may advance to full recognition or withdraw so quietly as to leave us in doubt, if indeed there had been any abberration from the normal conditions present in the case.

There can be no doubt that if the producing cause of the disturbance had never been in action, that the system should have remained in full play of complete harmony of function. Then if all disease in its incipiency be so occult as to be beyond our appreciation until it shall have made considerable advances toward dissolution of all or part, may we not be excusable for looking a little sharper and closer than the pathologists have hitherto been wont to look? It does seem to me that the true function of the philanthropic medical man in all the departments is not so much to study to be a mere mitigator of symptoms, as a preventor of the inception of disintegrating influences.

It has already been seen that the heat and swelling (upon which redness depends) owe their existence to a perverted molecular action. Then what are all these changes but a defective combination or separation of the chemical constituents of the body? or in other words, an interference with the normal play of affinities?

If, then, the whole matter of disease in general has been narrowed down to defective play of affinities, we are near the point to prescribe limits to this unfavorable condition of the molecules that compose our bodies. If, then, nutrition, inflammation, and all the vital changes occurring in living bodies, be but examples of burnings, oxidations, it clearly follows that the supply of fuel—oxidizable material—must have something important to do with not only disease, but health also; and brings us to the closing proposition, which is,—Proper food properly taken, exercise sufficient, and scrupulous cleanliness, will annihilate the use of all drugs and doses.

